# Supplementary material for: Broadband on-chip single-photon spectrometer
Source: Nat Commun. 2019 Sep 10;10:4104. doi: 10.1038/s41467-019-12149-x (PMC6736971; doi:10.1038/s41467-019-12149-x)
Supplement: Supplementary file 1 — Supplementary Information [file 41467_2019_12149_MOESM1_ESM.pdf]

Supplementary Information:
Broadband on-chip single-photon spectrometer

Risheng Cheng[1], Chang-Ling Zou[1,2], Xiang Guo[1], Sihao Wang[1], Xu Han[1], and Hong X. Tang[1,*]

[1]Department of Electrical Engineering, Yale University, New Haven, Connecticut 06511, USA.
[2]Department of Optics and Optical Engineering, University of Science and Technology of China, Hefei 230026, Anhui, China
[*]hong.tang@yale.edu

Supplementary Note 1: List of characterized devices

We have fabricated a total of 7 broadband devices, 2 telecom-band devices along with some component test structures on a single die of 12 mm × 20 mm. The radius of the Rowland circle is fixed at 400 µm for broadband design and 1.6 mm for telecom-band design. The blaze angle of the echelle grating for broadband devices are varied from 5 degree to 20 degree. In some of the broadband devices, two 60 nm-wide nanowires are connected in parallel as shown in Supplementary Figure 1a in order to boost the signal-to-noise ratio (SNR) compared to the single-nanowire design in Supplementary Figure 1b, while maintaining saturated internal detection efficiency[1–3]. All of the 9 spectrometer devices survived after fabrication and showed ∼10 µA I_{SW} in single-nanowire devices and ∼20 µA I_{SW} in double-nanowire devices, which are comparable with the test micron-length short wires, despite the substantially longer (1000×) meander nanowires fabricated. This implies the high quality and uniformity of our NbN film and holds promise for further scaling our devices without performance degradation. Supplementary Figure 1c shows the transmission electron micrograph (TEM) image of the nanowire cross-section. The 8 nm-thick NbN nanowire is covered by its native oxide layer, which is distinguished from slight colour difference and approximately 2 nm thick. See Methods section of the main text for more details on our fabrication method.

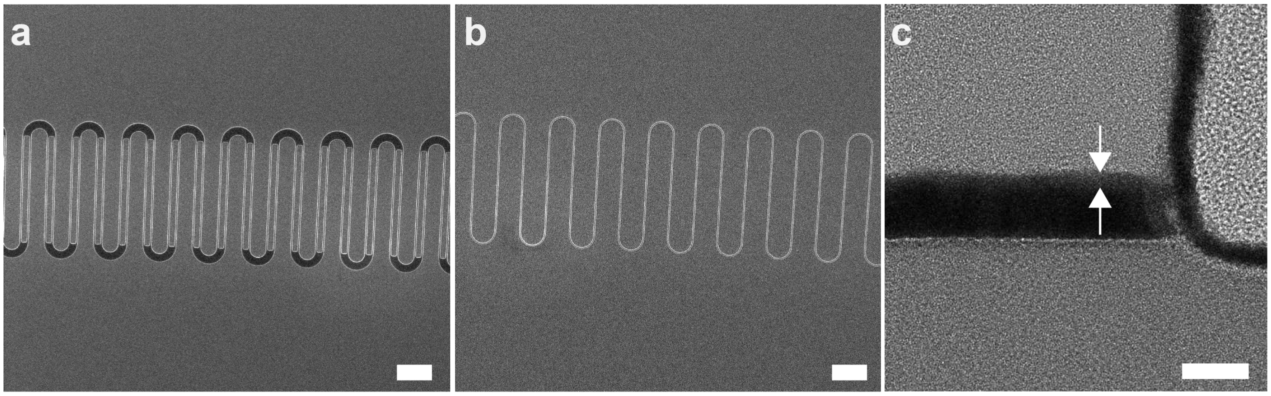

Supplementary Figure 1. (a,b) Close-up scanning electron micrograph (SEM) images of a section of the meander detector in broadband devices for (a) double-nanowire design and (b) single-nanowire design. The spacing between the parallel nanowire pairs is 150 nm. The width, pitch and depth of the nanowires are 60 nm, 700 nm and 3 µm, respectively. The images were taken prior to the deposition of high-*k* dielectric material and top ground electrode. Scale bar, 1 µm. (c) TEM image of the nanowire cross-section. The 8 nm-thick NbN nanowire is covered by its native oxide layer and HSQ etching mask. The oxide layer is approximately 2 nm thick and marked by a pair of arrows. Scale bar, 10 nm.

Supplementary Table 1 summarizes the design parameters and performance characterization results of all the prototype devices investigated in this work. The broadband design is based on a small grating pitch (0.8 µm) and fundamental diffraction mode (m=1) to minimize the order mixing. For example, the design does not support higher-order modes for 1000-2000 nm wavelengths, while second-order modes for 600-1000 nm wavelengths can be effectively suppressed by tuning the blaze angle of the echelle grating.

The trade-off of this design is that the zeroth-order mode is directly reflected without diffraction (see Figure 3a of the main text), resulting in a reduced detection efficiency. It is worth noting that for such broadband design covering both visible and IR wavebands, it is very difficult to obtain high diffraction efficiencies for the whole spectrum, since the blazing teeth of the grating in principle can only be optimized for a small range of wavelengths. However, this design can be adopted in the characterization of unknown light sources at single photon levels, such as fluorescence spectroscopy, where high efficiency is less critical than the operation bandwidth and spectral resolution.

	Device A	Device B	Device C	Device D
**Device type**	Telecom-band design	Broadband design	Broadband design	Broadband design
**Grating pitch**	8 μm	800 nm	800 nm	800 nm
**Rowland circle radius**	1600 μm	400 μm	400 μm	400 μm
**Blaze angle**	28 degree	15 degree	20 degree	15 degree
**Diffraction order number (*m*)**	6th	1st	1st	1st
**Bandwidth**	1420-1680 nm	400-2000 nm	400-2000 nm	400-2000 nm
**Nanowire width**	60 nm	60 nm	60 nm (double-nanowire)	60 nm (double-nanowire)
**Nanowire pitch**	700 nm	700 nm	700 nm	700 nm
**Nanowire depth**	3 μm	3 μm	3 μm	3 μm
**Total detector length**	2.62 mm	7.36 mm	7.36 mm	7.36 mm
**Detector covering angle**	8 degree	90 degree	90 degree	90 degree
**Total delay time**	2.355 ns	6.685 ns	4.625 ns	--
**Microwave propagation speed relative to vacuum speed**	0.74%	0.73%	1.06%	--
**Switching current**	10.4 μA	10.0 μA	19.7 μA	20.9 μA
**Spectral resolution**	< 2.5 nm	< 7 nm	4.4 - 6.6 nm	--
**Wavelength meter resolution**	0.1 nm @ 1550 nm	--	--	--
**System quantum efficiency (including all losses)**	0.1% @ 1550 nm	0.01% @ 1550 nm<br>0.05% @ 780 nm	0.05% @ 1550 nm<br>0.28% @ 780 nm	0.03% @ 1550 nm<br>0.18% @ 780 nm
**Reset time (1/e decay time)**	103 ns	220 ns	116 ns	116 ns
**Jitter**	40 ps FWHM @ 1550 nm	41 ps FWHM @ 1550 nm	--	--
**Dark count rate**	~6 Hz @ 90% Isw<br>~4 Hz @ 80% Isw	~30 Hz @ 90% Isw<br>~25 Hz @ 80% Isw	~6 Hz @ 90% Isw<br>~5 Hz @ 80% Isw	~4 Hz @ 90% Isw<br>~3 Hz @ 80% Isw

Supplementary Table 1. Summary of design parameters and performance characteristics for investigated devices.

In comparison, the telecom-band design uses much larger grating pitch (8 μm) and higher-order mode (*m*=6) to enhance the spectral resolution and the directivity at the expense of narrower operating bandwidth. Nevertheless, the results demonstrated in this work covers the whole S-, C-, L- and U-telecom wavebands, meeting requirements for most applications in quantum communications[4–7]. It is also relatively easy to optimize the device parameters so that high diffraction efficiencies could be achieved over 100-200 nm bandwidth[8,9].

For proof-of-principle demonstration, the efficiency of our devices is not optimal and limited by several imperfections which could be improved in future optimizations. Here, we estimate the main contributions to the loss of photons in Device A. Since these losses are wavelength dependent, we provide the estimated

loss range around 1550 nm as follows: diffraction efficiency loss (7-8 dB); loss due to incomplete nanowire absorption (8-9 dB); on-chip waveguide splitter loss (4-5 dB); fibre-to-waveguide coupling loss (6-7 dB); fibre-to-fibre coupling loss from multiple connectors inside and outside the cryostat (1.5-2 dB). Considering all these aspects, we estimate a typical total loss of the telecom-band device to be between 27-31 dB, which agrees with our measured system detection efficiency. By utilizing metal grating to replace the air grating (etched Si_3N_4) used in this work, the diffraction efficiency loss could be significantly reduced to < 1.5 dB as shown in Ref[9] and our simulation results. We also expect that the absorption efficiency of the NbN nanowires could be substantially enhanced to near-unity by the employment of wider, denser and longer nanowire detector structure (see the simulation results in Supplementary Note 4). The rest parts of the loss could be suppressed below 1.5 dB via splicing all fibres in the system (<0.1 dB loss), removing the on-chip waveguide splitter and the integration of the input waveguide with broadband spot-size converter[10]. Combined, we expect the whole system detection efficiency of our telecom-band device could reach around 50%.

Supplementary Note 2: Measurement set-up

Supplementary Figure 2 presents the schematic of the experimental set-up used for characterizing the spectrometer devices. The device chip was mounted on the millikelvin stage of a dilution refrigerator (BlueFors), which was working at 1.5 K mode to offer enough cooling power to operate the translation stages (Attocube). The DC bias current was applied to the superconducting nanowire through the DC ports of a pair of bias-tees, one of which was connected to a current source (Keithley 2401) while the other one was terminated by a 50 Ω resistor. In order to minimize the extra timing jitter introduced by the noise from readout electronics, we employed two SiGe cryogenic low-noise amplifiers operating at the 4 K stage of the cryostat and connected them to the RF ports of the bias-tees to read out the photon-excited detector pulses. We used either tunable lasers or broadband thermal light source along with a monochromator to measure the spectral response of our devices, by illuminating the device after an appropriate attenuation of the input power to single-photon level. More details on specific instruments used in the experiment are provided in the Methods section of the main text.

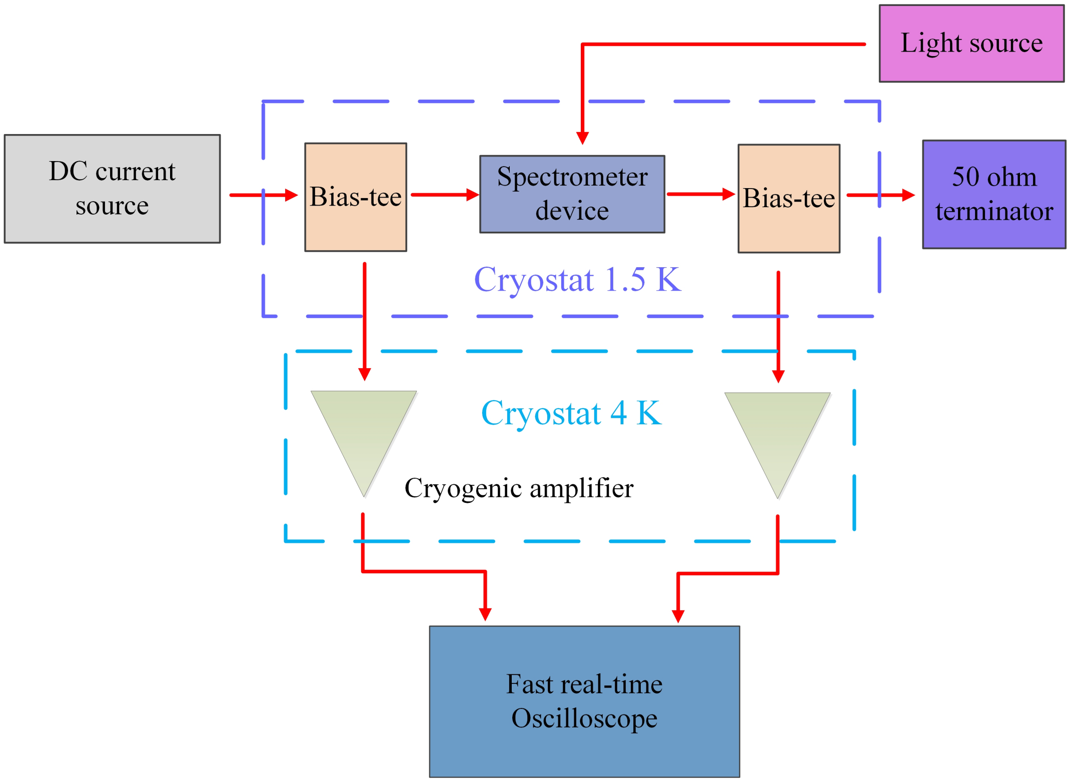

Supplementary Figure 2. Schematic of experimental set-up used for the device characterization.

Supplementary Figure 3 displays our cryogenic active side-coupling alignment set-up held at the millikelvin stage of the dilution refrigerator. The device chip containing multiple spectrometer devices was mounted on a 3-axis stack of Attocube stages (20 mm, 20 mm and 5 mm travelling range in XYZ direction, respectively) combined with a manual rotation stage. We drove the Attocube stages to move the chip and make

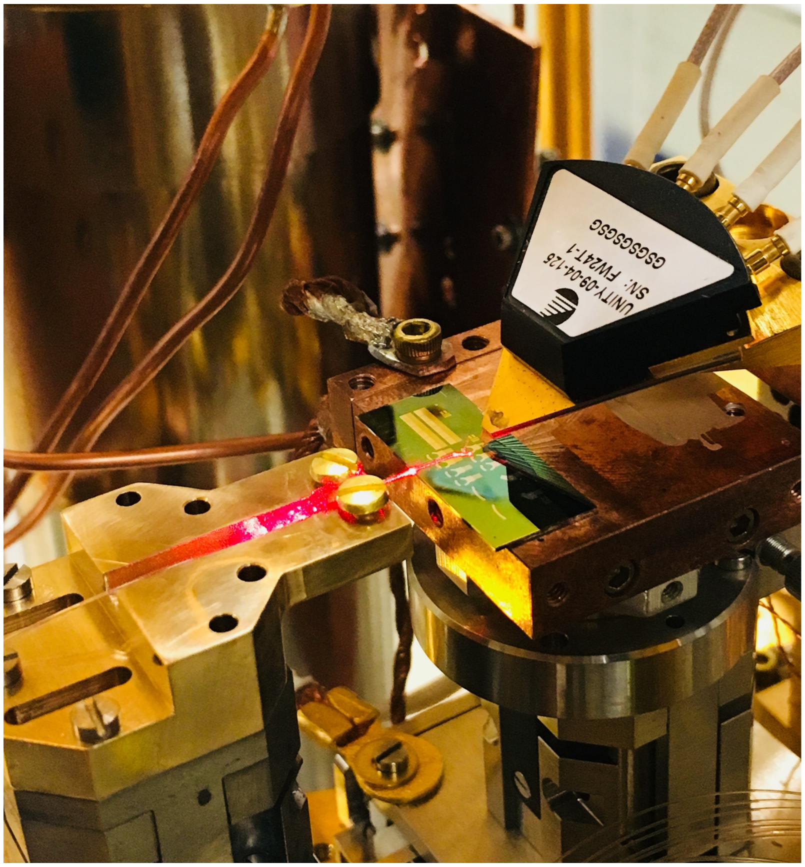

Supplementary Figure 3. Cryogenic active side-coupling alignment set-up.

electrical contact with a multi-channel RF probe for switching between devices. Another set of 3-axis Attocube stages (20 mm, 5 mm and 5 mm XYZ travelling range) were employed to align a single-mode tapered/lensed fibre in nanometre precision to couple light into the devices from the edge of the chip. This set-up not only guarantees broad band light coupling covering both visible and infrared wavebands but also allows for measurements on multiple devices in a single round of cool-down.

Supplementary Note 3: Microwave delay line design

Supplementary Figure 4a shows the cross-sectional schematic of the NbN superconducting nanowire delay line capped with 150 nm-thick alumina (AlO_x) and 150 nm-thick aluminium (Al). Owing to the high kinetic inductance of the NbN nanowire combined with the large capacitance introduced by the thin high-k (high dielectric constant) layer, a group velocity as low as $0.73\%\,c$ was experimentally obtained with 60-nm-wide nanowires ($1.06\%\,c$ for double-nanowire detector), where c denotes the speed of light in vacuum. The slight difference of the experimentally measured value from the numerically calculated result $0.58\%\,c$ (Supplementary Figure 4b) could be attributed to the remaining 80 nm-thick HSQ mask atop the NbN wire (not shown in the schematic). It is critical to cap the nanowire with dielectric spacing layer and metal top ground to form a transmission line. Without these structures, the propagation speed in the nanowire is much faster ($>10\%\,c$), and the cross-talk between neighboring nanowires is significant, resulting in seriously degraded spectral resolution of the spectrometer device.

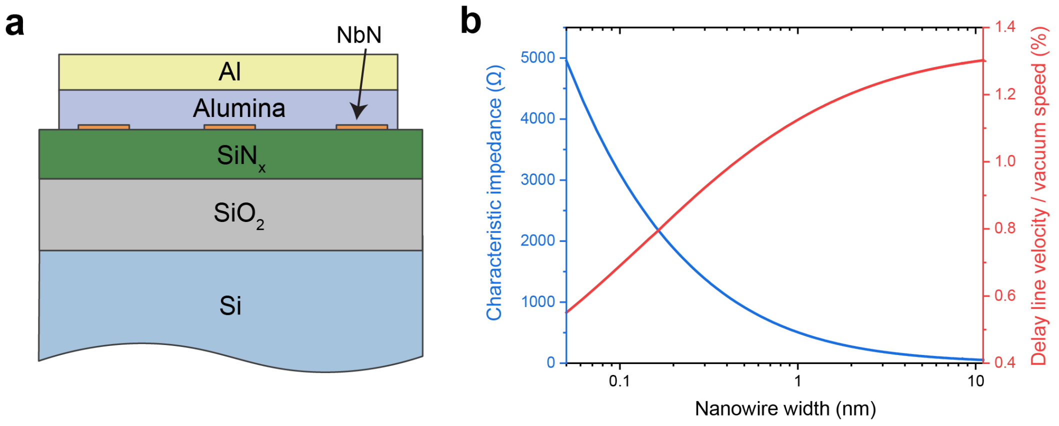

Supplementary Figure 4. (a) Cross-sectional schematic of the superconducting nanowire delay line. (b) Calculated characteristic impedance and relative propagation group velocity of the nanowire delay line as a function of the nanowire width. The calculation was based on 150 pH/square sheet kinetic inductance of the NbN wire which was measured separately.

Both ends of the nanowire delay line are gradually tapered to a width of 8 µm, while the characteristic impedance is transformed from several kΩ to 50 Ω to match the impedance of readout electronics. Each of the taper consists of 37 straight sections with a length of 220 µm and U-shaped bends connecting them in between (see Figure 2b of the main text). The radii of the bends were chosen so that the gaps between the straight sections are three times of the straight wire width or 10 µm, whichever are larger. Klopfenstein-type tapers were leveraged in our design and the required widths for each sections of the taper were derived from the calculated relationship of impedance and nanowire width shown in Supplementary Figure 4b. The total length of the resulting tapers is 8.8 mm, corresponding to 2.5 times wavelength of 1 GHz signal in the transmission line.

Supplementary Note 4: Simulation results of nanowire absorption

Supplementary Figure 5 shows the simulation results for the nanowire absorption rates, extracted from the solved effective index of the slab Si_3N_4 waveguide together with the NbN nanowire, AlO_x spacing layer and Al top ground layer placed on top (see the cross-section shown in Supplementary Figure 4a). Only TE modes were considered here. As shown in the results, the absorption rates peak at near-IR region around 800 nm, which roughly agrees with our raw data of the photon counts histogram (Supplementary Figure 10). The absorption rates decrease at shorter wavelengths due to weaker evanescent field, while the rates at longer wavelengths are limited by the finite penetration of the electrical field into the nanowire. The absorption could be significantly enhanced in the optimized design by using wider, denser and longer nanowires in conjunction with thicker AlO_x dielectric spacing layer. We also have recently experimentally confirmed that 100 nm-wide NbN nanowires used in the optimized design could still exhibit saturated efficiency at the wavelength of 1550 nm.

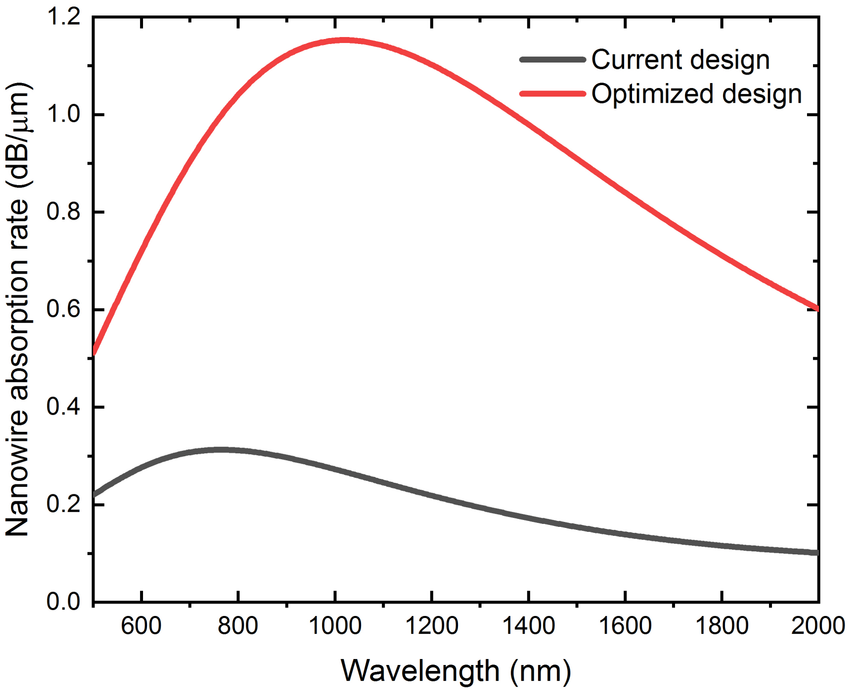

Supplementary Figure 5. Simulation results of the nanowire absorption rates as a function of the wavelength. The width and pitch of the nanowire were set to 60 nm and 700 nm in the current design while 100 nm and 400 nm in the optimized design. For further enhancement of the absorption, the thickness of the AlO_x layer was enlarged from 150 nm to 300 nm in the optimized design.

Supplementary Note 5: Spectral resolution

Theoretical analysis

As presented in the main text, the diffraction angle of input light is determined by

$$d(\sin(\theta_{\text{in}})+\sin(\theta_m))=\frac{m\lambda}{n_{\text{eff}}}, \tag{1}$$

where d is the period of the grating, θ_{in} is the angle of incidence, θ_m is the angle of the mth order diffraction, λ is the wavelength of the incident light in free space, and n_{eff} is the effective refractive index of the mode in the slab waveguide.

The corresponding spectral-to-spatial mapping gives

$$\Delta\theta_m=\frac{m}{dn_{\text{eff}}\cos(\theta_m)}\Delta\lambda. \tag{2}$$

By way of the spatial-to-temporal mapping, we have the arrival time difference

$$\Delta t=4R_{\text{Rowland}}\Delta\theta_m D_{\text{nw}}/P_{\text{nw}}/v_{\text{mw}}, \tag{3}$$

with R_{Rowland} the radius of the Rowland circle, D_{nw} the depth of the meander nanowire, P_{nw} the pitch of the nanowire and v_{mw} the group velocity of the microwave signal propagating along the nanowire delay line. Therefore, the spectral-to-temporal mapping sensitivity is provided by

$$\xi=\frac{\Delta t}{\Delta\lambda}=\frac{m}{dn_{\text{eff}}\cos(\theta_m)}\frac{4R_{\text{Rowland}}D_{\text{nw}}}{v_{\text{mw}}P_{\text{nw}}}. \tag{4}$$

The spectral resolution of the device δ_λ is limited by the finite size of the beam spot focused on the Rowland circle where the nanowire detector is mounted, due to the diffraction limit of light. The intensity distribution of the focused beam for monochrome light can be approximated by a Gaussian function with a FWHM value of δ_{beam}, which corresponds to $\delta_\theta=\frac{1}{2}\delta_{\text{beam}}/R_{\text{Rowland}}$. On the other hand, the timing jitter j_{noise} introduced by the noise of readout electronics also deteriorates the spectral resolution. Combining

with equation (2) and (4), we obtain

$$\delta_\lambda = \sqrt{[\frac{dn_{\text{eff}}\cos(\theta_m)}{m}]^2\delta_\theta^2 + \frac{j_{\text{noise}}^2}{\xi^2}} \tag{5}$$

The following are several comments concerning the sensitivity and resolution of the spectrometer design based on equation (4) and (5):

(1) In order to enhance the sensitivity and thus the spectral resolution, an echelle grating with a smaller grating period d, higher diffraction order mode with larger m and material with lower index for smaller n_{eff} are preferred.

(2) The spectral resolution of the spectrometer device δ_λ is linearly proportional to the diffraction angle resolution δ_θ, since the contribution of the noise induced jitter is negligible as will be discussed in the following subsection. Due to the limitation of δ_{beam} set by the light diffraction, δ_θ and δ_λ is finally determined by the radius of the Rowland circle or the footprint of the device. That is, the spectral resolution of the device could be enhanced by scaling to a larger device size. More detailed discussion on the further scalability compatible with microfabrication processes will be provided in the Section 7.

(3) The wavelength of input photons can be estimated according to the sensitivity ξ. As noted in the main text, there is no discrete pixel in our nanowire detectors, and thus the measurement imprecision is determined by the noise and uncertainties of the spectral-to-spatial and spatial-to-temporal mappings along with the electronic readout circuits. In practice, the measurement imprecision would be improved by repetitive measurement. For example, when N_c monochrome photons are counted by our spectrometer, the mean value of the Δt could be used for estimating the wavelength, with the imprecision reduced to $\delta_\lambda/\sqrt{N_c}$. This indicates that our device could also work as a wavelength-meter with a very high precision that is orders of magnitude smaller than the single-shot spectral resolution. As all the quantities (spatial or temporal variable) in our spectrometer are continuous variables, our device is significantly distinct from its conventional counterparts which rely on the multi-pixel detectors.

Electronic noise contribution to the timing jitter

Supplementary Figure 6 shows the measured slew rates of the photon-excited detector pulses for the broadband devices (Device B and Device C in Supplementary Table 1) with varying bias currents applied. The slew rates were measured by using a fast oscilloscope (Lecroy HDO9404) to take the derivatives on the acquired pulse waveforms at the discrimination threshold level in real-time. Each data points in the

figure are averaged results from multiple measurements. We could calculate the noise contribution to the jitter $j_{\rm noise}$ by the equation $j_{\rm noise} = \delta_{\rm noise}/SR$, where $\delta_{\rm noise}$ is the FWHM of the background electric noise distribution associated with the readout electronics and SR is the measured slew rate. $\delta_{\rm noise}$ was extracted by the Gaussian fitting to the experimentally measured histogram of the noise level taken by the oscilloscope after the amplification. In our measurement set-up, $\delta_{\rm noise}$ from both cryogenic amplifiers are 1.6 mV that is independent of bias currents and measured devices. Therefore, we estimate the total jitter introduced by the noise of reading electronics is less than 3 ps in FWHM for both of the devices when the bias current is applied higher than 80% of the switching current. This value is well below those extracted from histogram peaks (Supplementary Figure 8b), suggesting that the spatial mapping resolution of the superconducting nanowire delay line is limited by the size of focused beam spot on the nanowire detector rather than the electric noises.

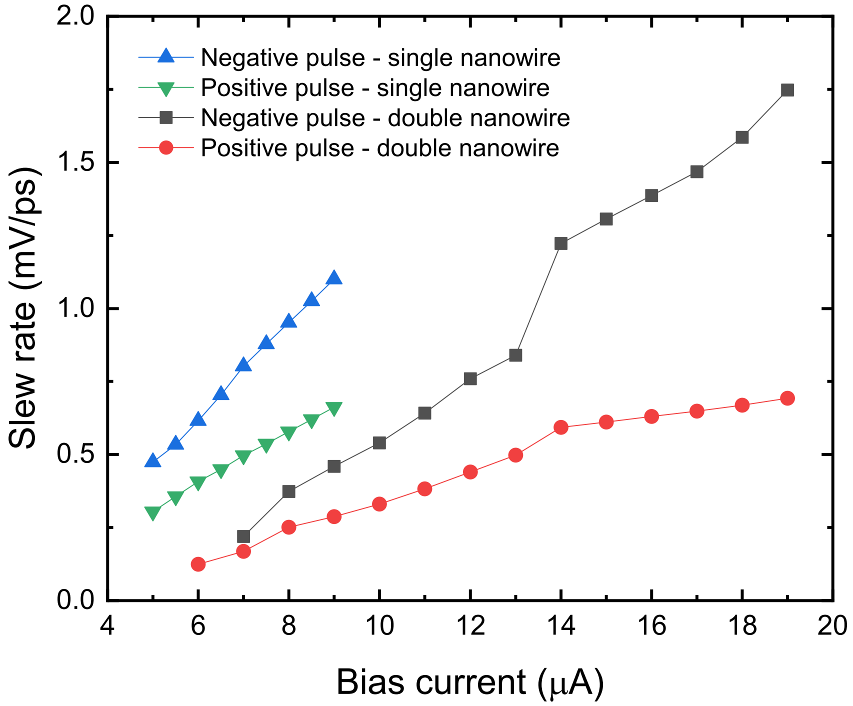

Supplementary Figure 6. Measured slew rates for broadband devices with single meander nanowire and double parallel nanowires (Device B and Device C). The slew rates for negative pulses are in absolute values. The slew rates for negative pulses are significantly larger than those for positive pulses due to the higher saturation level of the amplifiers.

Effect of bias current

Supplementary Figure 7 demonstrates the normalized histogram of photon counts as a function of the arrival time difference Δt taken from one of the telecom-band device (Device A in Supplementary Table 1)

with different bias currents applied. The switching current of the device is 10.4 µA. The curves gradually converge in high bias current region, where the nanowire detector is typically biased during the operation, which guarantees the stability of the results particularly when the spectrometer is operated in wavelength-meter mode for a long integration time. We attribute the deviation of the low bias results to the nonlinear response of the cryogenic amplifiers, which have different saturation levels depending on the polarity of the signal, despite that two amplifiers with an identical model were employed in our measurement.

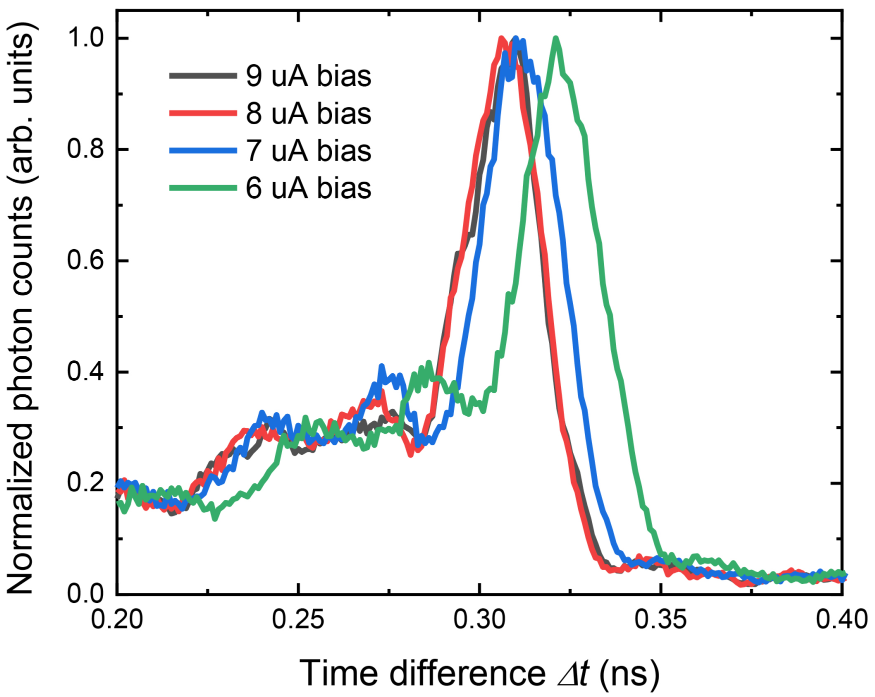

Supplementary Figure 7. Normalized histogram of photon counts versus arrival time difference Δt measured at different bias currents.

FWHM of arrival time distribution

Supplementary Figure 8 shows the zoom-in view of peaks and FWHM values extracted from the photon counting histogram measured with Device C (see Figure 3b in the main text). The spectral resolution of the device can be calculated by $\delta_\lambda = \delta_{\Delta t}/\xi$, where $\delta_{\Delta t}$ is the FWHM of the arrival time difference Δt shown in Supplementary Figure 8b, and the sensitivity ξ could be estimated based on the equation (4). The device shows better than 7 nm resolution over the whole spectrum and the best result 4.5 nm was recorded in the visible region. Based on $\delta_{\Delta t}$ and the calculated microwave propagation speed, we could estimate the size of the focused beam spot on the nanowire detector to be 1.7-2.5 µm, which agrees well with the finite-difference time-domain (FDTD) simulation results. The variation of the FWHM values

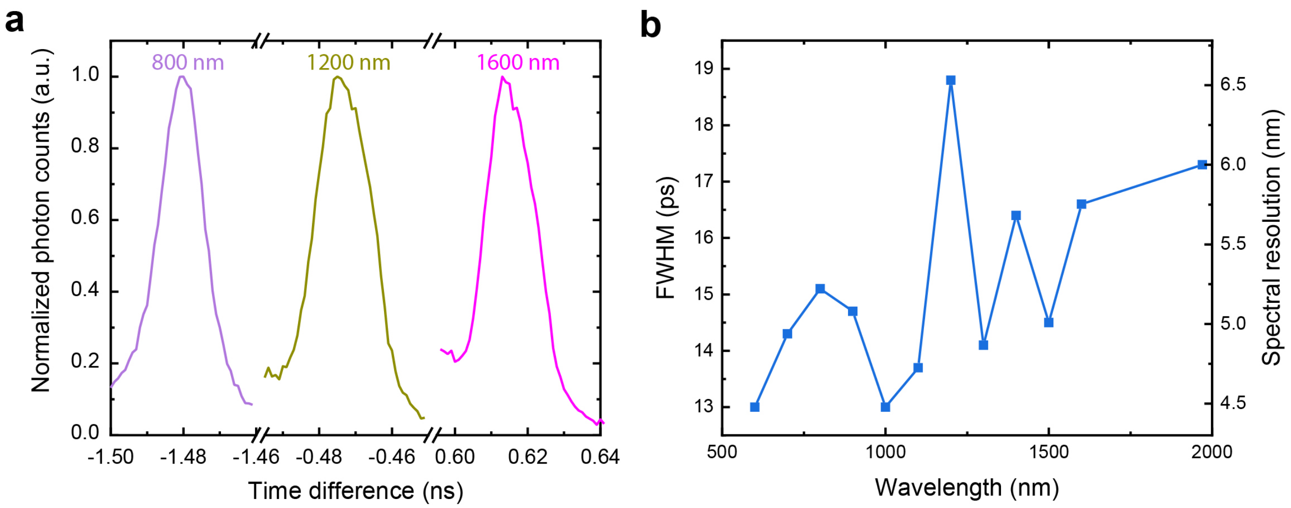

Supplementary Figure 8. (a) Zoom-in view of normalized photon counting histogram measured for different wavelength sources. (b) FWHM extracted from histogram peaks and the corresponding calculated spectral resolution. The results were obtained from one of the broadband device (Device C) with the nanowire detector biased at 80% of its switching current I_{SW}.

over the wavelength can be from the asymmetry of the histogram peaks, making the Gaussian fitting less trustworthy (see Supplementary Note 7 for more detailed discussion).

Supplementary Note 6: Timing jitter

Our on-chip spectrometer not only provides high spectral resolution to continuous-wave (CW) photon sources, but also responds to pulsed sources and simultaneously records the spectral and arrival timing information. We measured for Device C the histogram of photon counts as a function of the time difference Δt between two ends of the nanowire detector using 1550 nm CW laser and a 2.4 ps-pulsed 1560 nm laser (PolarOnyx) respectively for comparison. Supplementary Figure 9a shows similar distribution for both sources except for the slight difference in the central position of the peaks, owing to the narrower spectral bandwidth of the pulsed laser (~ 1.5 nm) in comparison with the resolution of the spectrometer (5-7 nm).

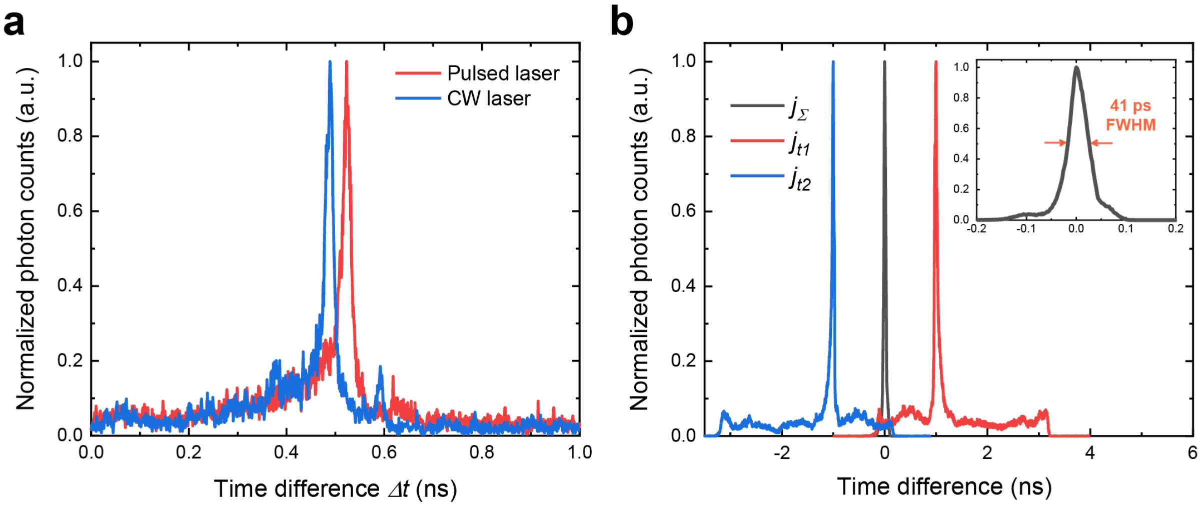

Supplementary Figure 9. (a) Normalized histogram of photon counts versus arrival time difference between signals from two ends of the detector. (b) Normalized histogram of photon counts versus arrival time difference between laser synchronization signal and detector signal. Inset shows the zoom-in view of j_{Σ}. The FWHM of the histogram profile is 41 ps, which defines the timing jitter of the device. The curves are appropriately offset in time domain for better visibility and comparison. All the histogram results were measured with the nanowire detector biased at 80% of I_{SW}.

To investigate the jitter performance of the device, we recorded the histogram of time difference between the laser synchronization signal and the pulses from each end of the detector (j_{t1} and j_{t2} in Supplementary Figure 9b). j_{Σ} is the calculated jitter distribution by averaging j_{t1} and j_{t2} to eliminate the geometry induced jitter[11], which shows slight improvement with 41 ps FWHM. We also measured the jitter for the telecom-band device (Device A) and obtained similar results (see Figure 4d in the main text).

Supplementary Note 7: Raw data of photon counts histogram

Supplementary Figure 10 shows the histogram of raw photon counts taken from the broadband device (Device C). The results for each wavelength were obtained by integrating the counts for 30 minutes using a fast real-time oscilloscope with 1 ps time bin. It should be noted that the relative height of the histogram peaks reflect the SNR for different wavelength sources that is defined by the ratio of diffracted photon number to that of scattered ones. Since the oscilloscope registers the detection events at a constant rate ($\sim$ 50 Hz in our measurement) irrelevant to the real counting rates of the device (up to several MHz), the information of the detection efficiency is missing in this measurement. We calibrated the system efficiency of our devices using a dedicated pulse counter (Stanford Research SR400) and the results are shown in Supplementary Table 1. For practical applications, a high-speed time correlator with picoseconds timing resolution should be used instead of an oscilloscope in order to collect all the detector pulses and thus significantly cut down the integration time.

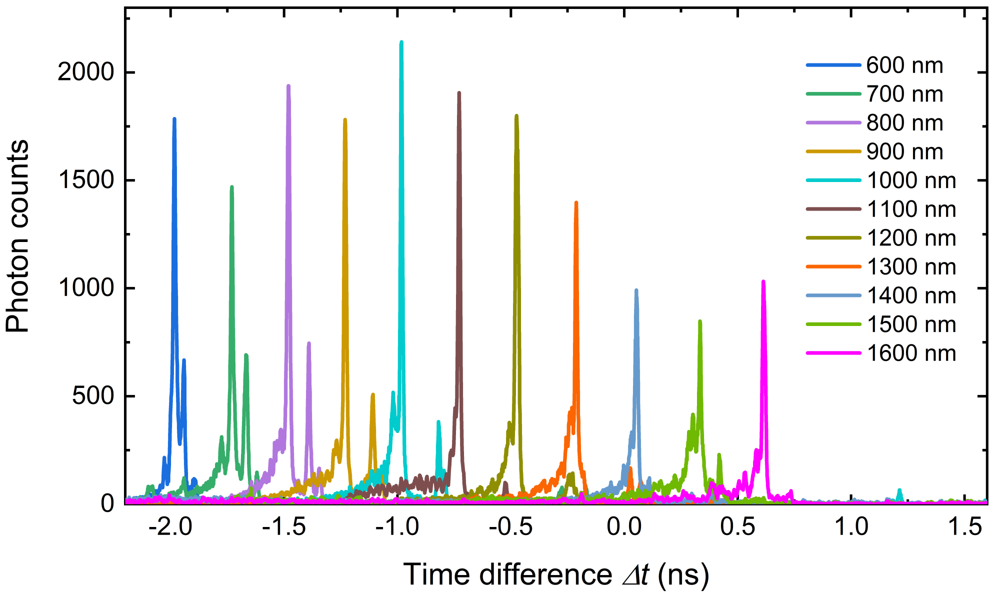

Supplementary Figure 10. Histogram of raw photon counts measured by fast oscilloscope versus time difference Δt measured for photon sources of different wavelengths. The histogram is recorded with the nanowire detector biased at 80% of its switching current I_{SW}.

We attribute the asymmetricity observed in the histogram peaks to the limited acceptance angle of the grating. For 1.2 µm input waveguide width used in this work, the divergence angle of the output light into the free propagation region is quite large, and thus the grating could not accept and reflect all power of the light. In particular, the power leakage from the left side of the grating is much more than right side, because the input waveguide is placed at the right side (see Figure 2a of the main text), resulting in the

asymmetrical long tails of the histogram peaks. We also note that the narrow waveguide width leads to a significant propagation loss in the waveguide for longer wavelength around 2000 nm due to the cut-off condition. In order to improve the SNR, we operated the nanowire detector at two-photon detection mode[1] (or arm-triggering mode) at lower bias current and orders of magnitude stronger intensity of the light, when we were performing the histogram measurement for 1970 nm wavelength line (see Figure 3b of the main text). Both of the problem could be addressed by adopting a wider input waveguide (4-5 μm width) without sacrificing too much resolving power, which was confirmed by our FDTD simulation results.

Supplementary Note 8: Further scalability

In this work, we used devices of relatively small footprint to obtain reasonable device fabrication yield and demonstrate the device operation principle. By scaling to larger Rowland circles and longer nanowires, we expect that the device spectral resolving power can be further enhanced. Supplementary Figure 11 displays the scaling trends of the telecom-band spectrometer design with varying nanowire length and Rowland circle radius. For a nanowire of fixed length, there shows a trade-off between the resolution and operation bandwidth. For example, the resolution can be enhanced by simply enlarging the grating size but at the expense of the reduced bandwidth due to the smaller angle to be covered by the nanowire. It can be seen that it is feasible to realize 100 pm resolution with 200 nm bandwidth and thus 2,000 wavelength channels with a device based on 50 mm-radius Rowland circle and 40 mm-long nanowire. The calculation was done based on equation (4) and (5), assuming the focused beam spot size to be 4 µm.

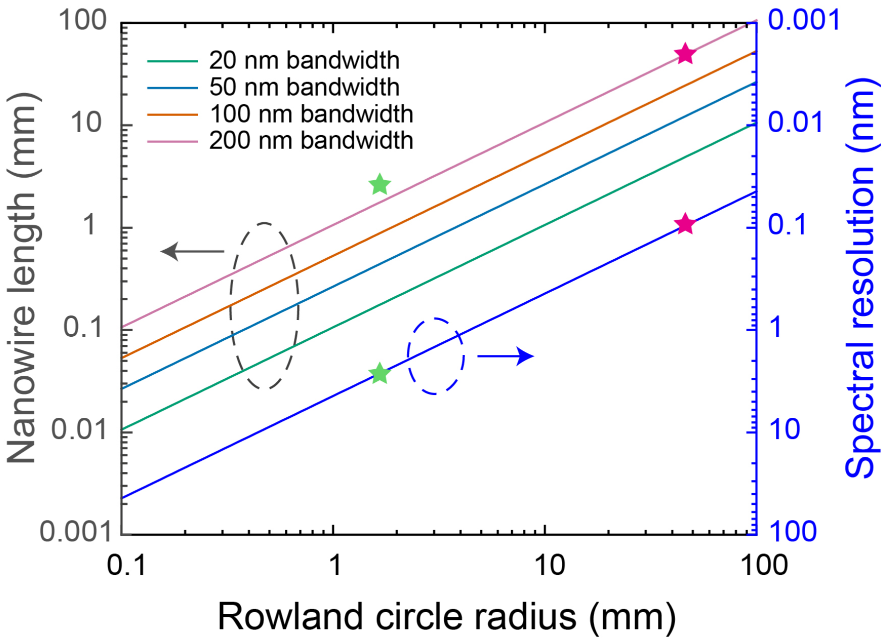

Supplementary Figure 11. Calculated spectral resolution and required length of the nanowire for the telecom-band design with a given Rowland circle radius. The magenta star symbols mark the design parameters and the expected resolution for scaled devices, while the green ones stand for the current device measured in this work.

Supplementary References

1. Marsili, F. *et al.* Single-photon detectors based on ultranarrow superconducting nanowires. *Nano Lett.* **11**, 2048–2053 (2011).
2. Cheng, R., Poot, M., Guo, X., Fan, L. & Tang, H. X. Large-area superconducting nanowire single-photon detector with double-stage avalanche structure. *IEEE Transactions on Appl. Supercond.* **27**, 1–5 (2017).
3. Cheng, R. *et al.* Self-aligned multi-channel superconducting nanowire single-photon detectors. *Opt. Express* **24**, 27070–27076 (2016).
4. Ciurana, A. *et al.* Quantum metropolitan optical network based on wavelength division multiplexing. *Opt. Express* **22**, 1576–1593 (2014).
5. Dynes, J. F. *et al.* Ultra-high bandwidth quantum secured data transmission. *Sci. Reports* **6**, 35149 (2016).
6. Wengerowsky, S., Joshi, S. K., Steinlechner, F., Hübel, H. & Ursin, R. An entanglement-based wavelength-multiplexed quantum communication network. *Nature* **564**, 225 (2018).
7. Eriksson, T. A. *et al.* Wavelength division multiplexing of continuous variable quantum key distribution and 18.3 tbit/s data channels. *Commun. Phys.* **2**, 9 (2019).
8. Sciancalepore, C. *et al.* Low-crosstalk fabrication-insensitive echelle grating demultiplexers on silicon-on-insulator. *IEEE Photon. Technol. Lett* **27**, 494–497 (2015).
9. Xie, S., Meng, Y., Bland-Hawthorn, J., Veilleux, S. & Dagenais, M. Silicon nitride/silicon dioxide echelle grating spectrometer for operation near 1.55 μm. *IEEE Photonics J.* **10**, 1–7 (2018).
10. Jia, L. *et al.* Mode size converter between high-index-contrast waveguide and cleaved single mode fiber using sion as intermediate material. *Opt. express* **22**, 23652–23660 (2014).
11. Calandri, N., Zhao, Q.-Y., Zhu, D., Dane, A. & Berggren, K. K. Superconducting nanowire detector jitter limited by detector geometry. *Appl. Phys. Lett.* **109**, 152601 (2016).
